# Sex-based differences in natural killer T cell-mediated protection against diet-induced steatohepatitis in Balb/c mice

**DOI:** 10.1186/s13293-023-00569-w

**Published:** 2023-11-14

**Authors:** Carlos Cuño-Gómiz, Estefanía de Gregorio, Anna Tutusaus, Patricia Rider, Nuria Andrés-Sánchez, Anna Colell, Albert Morales, Montserrat Marí

**Affiliations:** 1 Department of Cell Death and Proliferation, IIBB, CSIC, IDIBAPS, 08036 Barcelona, Spain; 2 Departament de Biomedicina, Facultat de Medicina, Universitat de Barcelona, 08036 Barcelona, Spain; 3 Institute of Molecular Genetics of Montpellier (IGMM), University of Montpellier, CNRS, INSERM, 34293 Montpellier, France

**Keywords:** Metabolic dysfunction-associated steatotic liver disease (MASLD), Metabolic dysfunction-associated steatohepatitis (MASH), Non-alcoholic fatty liver disease (NAFLD), Non-alcoholic steatohepatitis (NASH), Sex differences, Hepatic NKT cells, CD1d, Liver, Inflammation

## Abstract

**Background:**

Metabolic dysfunction-associated steatotic liver disease (MASLD) is prevalent in Western countries, evolving into metabolic dysfunction-associated steatohepatitis (MASH) with a sexual dimorphism. Fertile women exhibit lower MASLD risk than men, which diminishes post-menopause. While NKT-cell involvement in steatohepatitis is debated, discrepancies may stem from varied mouse strains used, predominantly C57BL6/J with Th1-dominant responses. Exploration of steatohepatitis, encompassing both genders, using Balb/c background, with Th2-dominant immune response, and CD1d-deficient mice in the Balb/c background (lacking Type I and Type II NKT cells) can clarify gender disparities and NKT-cell influence on MASH progression.

**Methods:**

A high fat and choline-deficient (HFCD) diet was used in male and female mice, Balb/c mice or CD1d^−/−^ mice in the Balb/c background that exhibit a Th2-dominant immune response. Liver fibrosis and inflammatory gene expression were measured by qPCR, and histology assessment. NKT cells, T cells, macrophages and neutrophils were assessed by flow cytometry.

**Results:**

Female mice displayed milder steatohepatitis after 6 weeks of HFCD, showing reduced liver damage, inflammation, and fibrosis compared to males. Male Balb/c mice exhibited NKT-cell protection against steatohepatitis whereas CD1d^−/−^ males on HFCD presented decreased hepatoprotection, increased liver fibrosis, inflammation, neutrophilic infiltration, and inflammatory macrophages. In contrast, the NKT-cell role was negligible in early steatohepatitis development in both female mice, as fibrosis and inflammation were similar despite augmented liver damage in CD1d^−/−^ females. Relevant, hepatic type I NKT levels in female Balb/c mice were significantly lower than in male.

**Conclusions:**

NKT cells exert a protective role against experimental steatohepatitis as HFCD-treated CD1d^−/−^ males had more severe fibrosis and inflammation than male Balb/c mice. In females, the HFCD-induced hepatocellular damage and the immune response are less affected by NKT cells on early steatohepatitis progression, underscoring sex-specific NKT-cell influence in MASH development.

**Supplementary Information:**

The online version contains supplementary material available at 10.1186/s13293-023-00569-w.

## Plain language summary

Metabolic dysfunction-associated steatotic liver disease (MASLD) is a common liver condition today. In its more advanced form, called metabolic dysfunction-associated steatohepatitis (MASH), adult men are more often affected than women, though this difference vanishes after menopause. Various factors contribute to MASH, including a specific immune cell type called NKT cells, which has not been deeply researched yet. To explore the role of NKT cells in steatohepatitis, we used male and female mice with or without NKT cells (CD1d^−/−^ mice), feeding them a high-fat diet that induces steatohepatitis. Our findings revealed that female mice had less severe steatohepatitis compared to males. Interestingly, we observed a protective role of NKT cells during steatohepatitis, as male mice without these cells had more damage, inflammation, and fibrosis than those with NKT cells. However, in females, even though those lacking NKT cells showed more liver damage and immune alterations, NKT did not seem to play a major role in early steatohepatitis progression. Notably, females had much fewer NKT cells in their livers compared to males, possibly explaining this difference. In conclusion, NKT cells seem to slow down steatohepatitis progression, especially in male mice. In females, their impact on early steatohepatitis advance appears more limited.

**Supplementary Information:**

The online version contains supplementary material available at 10.1186/s13293-023-00569-w.

## Highlights

- Steatohepatitis induced by a high-fat choline-deficient diet (HFCD) progresses faster in male Balb/c mice compared to females.
- Male CD1d^−/−^ mice exhibited exacerbated liver damage, inflammation, and fibrosis in response to the MASH diet, as compared to Balb/c male mice.
- The absence of NKT cells in males led to an altered immune landscape characterized by elevated neutrophil and inflammatory macrophage presence in MASH mice.
- Hepatic iNKT cell levels in female Balb/c mice were significantly lower than those in their male counterparts, and the absence of NKT cells sensitized females to a lesser extent than in male mice after HFCD feeding.
- Noteworthy sex disparities were observed in inflammatory cytokines, NKT cell-derived gene expression, and immune cell profiles, impacting the immune response during steatohepatitis progression.

**Supplementary Information:**

The online version contains supplementary material available at 10.1186/s13293-023-00569-w.

## Background

Natural killer T cells (NKTs), first named in 1995 [1], are a heterogeneous group of non-conventional T cells that express both natural killer (NK) cells and lymphocytes T markers, presenting characteristics of both the adaptive and the innate immune system [2, 3]. Based on the expression of the TCR, these cells can be subdivided into two subpopulations: classic NKTs, also known as type I or invariants (iNKTs), which express a semi-invariant T cell receptor (TCR) [4]; and non-classic NKTs or type II, which express a broader TCR repertoire [5].

Unlike conventional T cells, which recognize peptide antigens through the type I and II major histocompatibility complex (MHC), NKTs recognize lipid antigens presented by the non-classical MHC type I, CD1d [6], and can be activated by both self-antigens and foreign/microbial antigens [7]. In the liver, CD1d is expressed on Kupffer cells (KCs), hepatic stellate cells (HSCs), hepatocytes, circulating dendritic cells (DCs) and endothelial cells of the hepatic sinusoids, where NKT cells patrol [8].

NKT cells have been detected in numerous tissues, such as thymus, spleen, lung, bone marrow, lymph nodes, intestines, blood and liver, the latter being the one with the highest frequency of NKTs [9], 20- to 100-fold higher than in other organs [6]. In the murine liver, NKTs represent 20–30% of the total population of hepatic lymphocytes, while in the case of humans, this percentage is reduced to < 1% of hepatic lymphocytes [10, 11]. Of note, while in the murine model liver type I iNKTs are much more abundant than type II NKTs, in humans, the situation is the opposite [10, 12].

Similar to what happens with other cells of the innate immune system (such as macrophages, NK cells or neutrophils), NKTs respond quickly to stimuli capable of activating them, secreting a broad profile of effector cytokines and chemokines in large quantities [13], that can be divided into NKT1 (IFNγ), NKT2 (IL4) or NKT17 (IL17) cytokines. The NKTs will secrete different cytokine profiles based on the tissue milieu, the antigen-presenting cell and the type of lipid antigen [14]. Additionally, distinct subsets of NKTs tend to be more prevalent in different tissues [15]. In particular, NKT1 cells are the most abundant hepatic subset in C57BL/6 mice, while Balb/c mice presents a majority of hepatic NKT2 cells [15], indicating that there can be also strain-dependent differences in NKT-dependent effector properties. Finally, activated iNKTs can unfold perforin-dependent and FasL-dependent cytotoxic functions [16].

Due to the wide repertoire of cytokines that NKTs are capable of secreting, these cells have been implicated in numerous immune responses, including those against infectious agents [17, 18] and tumors [19], regulating multiple autoimmune [20] and inflammatory diseases [21]. In the liver, the activation of NKTs seems to contribute both to the onset and to the end of the inflammation and fibrosis, depending on the liver disease being evaluated, generating much controversy in this regard [14].

Metabolic dysfunction-associated steatotic liver disease (MASLD), previously known as non-alcoholic fatty liver disease (NAFLD) [22], defined by the presence of hepatic steatosis without causes for secondary hepatic fat accumulation [23], is the most common liver disease in Western countries, has a global prevalence of 25%, depending on the method of diagnosis, age, sex and ethnicity [24, 25]. To date, there are no approved drugs for MASLD, so given the rising global burden of MASLD and its economic impact there is an urgent need to understand the cellular and molecular mechanisms responsible for disease progression [26–28]. MASLD includes different histological conditions: simple fatty liver, characterized by the absence of hepatocellular damage, and MASH (metabolic dysfunction-associated steatohepatitis), characterized by the presence of inflammation and hepatocellular damage, with or without fibrosis. MASLD, and particularly MASH, can progress to cirrhosis and carcinoma hepatocellular [29]. For reasons that are not yet fully clarified, incidence and prevalence of MASLD is higher in men than in premenopausal women (age ≤ 50‐60 years), while after menopause MASLD occurs at higher rate in women [30].

Regarding research concerning NKT cells in metabolic diseases various studies have reported an aggravating role for NKT cells, for example in the liver of leptin-deficient *ob/ob* mice [31], high-fat diet (HFD) or obesity induced inflammation [32, 33], ethanol-induced liver damage [34], or steatohepatitis [35–38]. Some of these studies also observe that, in the most advanced stage of steatohepatitis, NKTs appear to accumulate in the liver promoting fibrosis by activating hepatic stellate cells [37, 38]. In contrast, other authors have reported opposite results as iNKT-cell-deficient mice displayed increased liver fibrosis in HFD-fed mice [39], suggesting a protective role for iNKTs in this setting. Given that NKT cells are at the crossroad of adaptive and innate immune system, it is possible that some of these discrepancies are due to the genetic background of the mice used. Worth mentioning, the studies that support a detrimental role for NKT cells in steatohepatitis were performed in C57Bl6/J mice [32–38], with a Th1-dominant immune response. However, the study of Miyagi et al. [39], in which a protective role for iNKT-cells was seen, was performed using Jα18 deficient mice (lacking only type I NKTs) in the Balb/c strain, with a Th2-dominant response [40].

Thus, given the as yet unclear role of NKTs in MASLD/MASH, we wanted to shed some light on the matter by investigating the role of these cells in a mouse model of diet-induced steatohepatitis. To validate if the Th2-phenotype affected NKT cell behavior during MASH we chose to use the Balb/c strain and CD1d-deficient mice, in the Balb/c background. CD1d-deficient mice, lacking both type I and type II NKTs, the latter type more abundant in human than in mice [12], was the model selected as previous studies had been carried out in Balb/c Jα18-deficient mice lacking only type I NKTs, and the possible contribution of type II NKTs in this context had not been yet addressed [39].

Furthermore, taking into account that, to date, most of the studies have been performed in male mice and, to our knowledge, no comparative gender study of steatohepatitis with the Balb/c strain has been carried out in mice lacking NKTs, we also included females to assess the relevance of the genetic background and the existence of a possible sexual dimorphism in the role of NKT cells in this pathology.

Consistent with the Th2 immune response observed in the Balb/c strain, we hypothesize that NKT-cell-deficient mice will exhibit more aggravated steatohepatitis than their wild type counterparts. Females are also expected to have a milder form of steatohepatitis than males, consistent with the known protection provided by estrogens [41–43], and as it has been already reported by others that gender and the mouse strain affect MASLD [44, 45].

## Materials and methods

### Animal care and experimental protocol

Animal studies were approved by the institutional animal care committee (Universitat de Barcelona, protocol 177/20), in accordance with the principles and procedures outlined in the National Institutes of Health Guide for the Care and Use of Laboratory Animals. Male and female Balb/c mice and CD1d knock-out mice (CD1d^−/−^), also in Balb/c background, were used at 6 to 8 weeks of age. Balb/c mice were purchased from Charles River, and the CD1d^−/−^ colony (The Jackson Laboratory, Stock #003814, C.129S2Cd1^tm1Gru^/J) was a gift from Drs Engel and Puñet-Ortiz, Universitat de Barcelona. All mice were housed in the animal facilities of the Faculty of Medicine (Universitat de Barcelona) with a 12-h light/dark cycle. Food and water were provided ad libitum*.* To study the role of NKT cells in a MASH model, animals were fed a high-fat choline-deficient diet (HFCD) (L-amino acid diet with 60% kcal from fat, 0.1% of methionine and no added choline, Open Source diets #A06071302) for 6 weeks [46]. Control animals were fed with a standard chow diet (CHOW). Mice, Balb/c and CD1d^−/−^, were divided by sex and genotype and randomly assigned into groups according to the following scheme: male mice [CHOW CD1d KO, n = 8; CHOW Balb/c, *n* = 8; HFCD CD1d KO, *n* = 10; HFCD Balb/c, *n* = 10]; female mice [CHOW CD1d KO, *n* = 8; CHOW Balb/c, *n* = 8; HFCD CD1d KO, n = 10; HFCD Balb/c, *n* = 10]. After 6 weeks of CHOW or HFCD diet, animals were killed, using pentobarbital (50 mg/kg) intraperitoneal injection, and blood and hepatic samples were obtained for analysis.

### NAFLD activity score (NAS)

NAS score was determined in H&E samples as previously reported [47]. In brief, NAS score was blindly assessed in CHOW and HFCD groups (*n* = 8 for CHOW groups, and *n* = 10 for HFCD groups) evaluating the degree of steatosis (ranging from 0 to 3 as follows: < 5% Score 0, 5%-33% Score 1, 33%− 66% Score 2, > 66% Score 3), lobular inflammation (ranging from 0 to 3 as follows: no foci Score 0, > 2 foci per 200X field Score 1, 2–4 foci per 200X filed Score 2, > 4 foci per 200X field Score 3), and ballooning (ranging from 0 to 2 as follows: no ballooning Score 0, few balloon cells Score 1, many cells/prominent ballooning Score 2). According to this algorithm, total NAS score ranges from 0 to 8 [47].

### RNA isolation and RT-PCR

Total RNA was isolated from the liver left lobe with TRIzol™ Reagent (Invitrogen, Carlsbad, CA, USA), following the manufacturer’s protocol (*n* = 5 for all groups). Reverse-transcription was performed with iScript cDNA Synthesis Kit (Bio-Rad Laboratories, Hercules, CA, USA) from 1 µg of RNA. Real-Time PCR was executed with iTaq Universal SYBR Green Supermix (Bio-Rad Laboratories) from 10 ng of cDNA, following the manufacturer’s instructions using an iCycler Thermal Cycler iQ5 Multicolor Real-Time PCR Detection System (Bio-Rad Laboratories). β-actin was used as a housekeeping gene since its expression was not affected by genotype, sex, or diet.

The primers sequences used were the following:

**Table:** 

Gene	Forward 5′ 3’	Reverse 5′ 3’
α-SMA	ATG GCT CTG GGC TCT GTA AG	CCC ATT CCA ACC ATT ACT CC -
β-Actin	GAC GGC CAG GTC ATC ACT AT	CGG ATG TCA ACG TCA CAC TT
CD1d	AAT TAC ACC TTC CGC TGC C	CTT CGT GAA GCT GAT GGT GG
Col1a1	GAG CGG AGA GTA CTG GAT CG	GTT CGG GCT GAT GTA CCA GT
F4/80	TTT CCT CGC CTG CTT CTT C	CCC CGT CTC TGT ATT CAA CC
IFN-γ	CAG CCA AGC GGC TGA CTG AA	GTG CTG TCT GGC CTG CTG TT
IL-1β	GAG CTG AAA GCT CTC CAC CTC	CTT TCC TTT GAG GCC CAA GGC
IL-4	CAG GAG AAG GGA CGC CAT GC	TGC GAA GCA CCT TGG AAG CC
IL-6	CCG GAG AGG AGA CTT CAC AG	CCG GAG AGG AGA CTT CAC AG
IL-10	CGG GAA GAC AAT AAC TGC ACC C	CGG TTA GCA GTA TGT TGT CCA GC
IL-12	ATA AGA CGA CGG CAC GAA GG	GGT AAG GAT GAA GAG GGA GTT CAA
CCL2	CAA GAA GGA ATG GGT CCA GA	GCT GAA GAC CTT AGG GCA GA
TNF-α	CTG AAC TTC GGG GTG ATC GGT	ACG TGG GCT ACA GGC TTG TCA

### Tissue analysis: H&E and Sirius red staining

A piece of the liver left lobe was formalin-fixed for 24 h and paraffin embedded. Sections of 7 µm were routinely stained with H&E or 0.1% Sirius red-picric solution following standard procedures (*n* = 8 for CHOW groups, and *n* = 10 for HFCD groups). The slices were examined with a Nikon (Tokyo, Japan) Eclipse E-1000 microscope equipped with an Olympus (Tokyo, Japan) DP72 camera. The quantification of the collagen fibers stained with the Sirius Red solution was blindly assessed in all the experimental groups with the ImageJ 1.48v software (National Institutes of Health, Bethesda, MD).

### Hydroxyproline quantification

Hepatic hydroxyproline levels, an indirect measure of collagen content, were determined (*n* = 8 for CHOW groups, and *n* = 10 for HFCD groups) by a modification of the protocol published by Reddy et al. [48]. Briefly, liver samples and trans-4-Hydroxy-L-proline standards were homogenized in 6N HCl and later hydrolyzed by autoclaving at 121°◦C for 25 min. Hydroxyproline content from samples and standards was then oxidized with Chloramine-T and colored with Ehrlich reagent; absorbance was read at 550 nm in a Multiskan Sky Microplate Spectrophotometer (ThermoFisher Scientific, Waltham, MA, USA).

### Transaminases, triglycerides and cholesterol

A blood sample was obtained from the animals at the time of killing. After a centrifugation (10,000*g*, 10 min, 4 °C), the serum was separated diluted 1/8 in saline (0,9% NaCl). Alanine and aspartate transaminases (ALT and AST) were measured (*n* = 8 for CHOW groups, and *n* = 10 for HFCD groups) using a biochemical analyzer at the Hospital Clinic, Barcelona.

A piece of liver (approximately 20 mg) obtained after killing was homogenized manually, with a disposable pellet pestle, in physiological saline. After centrifugation (10,000*g*, 10 min, 4 ºC), the supernatant was diluted 1/4 in physiological serum. Triglycerides and cholesterol (*n* = 8 for CHOW groups, and *n* = 10 for HFCD groups) in diluted homogenate were measured using a biochemical analyzer at the Hospital Clinic, Barcelona.

### Sodium dodecyl sulfate protein gel electrophoresis and immunoblot analysis

Cell lysates were prepared in RIPA buffer (50-mM Tris·HCl, pH 8, 150-mM NaCl, 1% Nonidet P-40, 0.1% sodium dodecyl sulfate, 1% Triton X-100 plus proteinase inhibitors). Protein concentration was determined by Bradford assay, and samples containing 10–50 μg were separated by sodium dodecyl sulfate protein gel electrophoresis. Proteins were transferred to nitrocellulose membranes. After this, membranes were blocked in 8% nonfat milk in 20-mM Tris–HCl, 150-mM NaCl, and 0.05% Tween 20 for 1 h at room temperature. Primary antibodies against α-SMA (clone 1A4), anti-β-actin-HRP (#A5228 and #A3854, respectively; Sigma-Aldrich), and anti-COL1A1 (#AB765P, Chemicon) were used.

### Immunohistochemical (IHC) staining

Formalin-fixed paraffin-embedded tissues, from HFCD Balb/c (n = 5) or HFCD CD1d^−/−^ (*n* = 4) male mice, were sliced into 4-µm sections. Following deparaffinization with xylene and decreasing concentrations of ethanol, antigen retrieval was performed with 10 mM citrate buffer (pH 6) at 100°C for 15 min. Then, endogenous peroxidase was blocked using 3% H_2_O_2_ in methanol and non-specific binding sites were blocked using 10% normal goat serum (#50197Z; ThermoFisher Scientific, Waltham, MA, USA). Slices were incubated overnight with unconjugated primary antibodies against MPO (#ab9535; Abcam, Cambridge, MA, USA) or F4/80 (#70076; Cell Signaling Technology, Danvers, MA, USA), 45 min with a biotinylated anti-rabbit IgG antibody (sc-2491, Santa Cruz Biotechnology, Santa Cruz, CA, USA) and 30 min with an ABC peroxidase complex (#32020, ThermoFisher Scientific). Finally, antigens were stained with DAB-peroxide buffer and tissue was counter-stained with hematoxylin and mounted with Aquatex (Merck Millipore, Burlington, MA). Images were visualized with a Nikon (Tokyo, Japan) Eclipse E-1000 microscope equipped with an Olympus (Tokyo, Japan) DP72 camera.

### Flow cytometry: preparation of cell suspension and antibodies

Mice were anesthetized with pentobarbital (50mg/kg, i.p.), the ventral midline abdomen and peritoneal wall opened to expose the liver, portal and cava veins. 100 ul of heparin (stock 1000U/mL in PBS + 10mM EDTA) were carefully injected in the inferior cava vein to avoid coagulation, a 24G catheter was placed and ligated into the portal vein and another 18G catheter in the inferior cava. Sutures were placed around the superior vena cava and close to the kidneys. The livers were perfused with 100 mL PBS + 10mM EDTA), the sutures closed, at a flow of 4ml/min, and the flow out was collected in a 100mL container placed under the 18G catheter. Afterwards, it was centrifuged at 600g, 5 min, the pellet resuspended in 1mL ACK lysis buffer (ThermoFisher #A1049201) for 5min at RT. Then, diluted with 20ml PBS + 2%FBS + 0.05%NaN3, and centrifuge again at 600g, 5 min. The clean pellet was then resuspended in PBS and cells were counted in hematocytometer. The cellular concentration was adjusted to approximately 1 × 10^6^ cells per condition in 100µl wash buffer (PBS + 2% FBS + 0.05% NaN3) including the antibodies used. Samples were incubated in the dark, 30 min, 4ºC, and afterwards washed in wash buffer (200µl), centrifuged at 600g, 5min, and resuspended in 100µl PBS. The antibodies used were: mouse CD3 FITC Conjugate #HM3401 (Invitrogen), mouse CD4 APC Conjugate #MCD0405 (Invitrogen), rat Anti-Mouse CD19-PE/CY5.5 #1575-16 (Southern Biotech), rat Anti-Mouse Ly6G/GR-1-PE/CY7 #1900–17 (Southern Biotech), Alexa Fluor 700 Rat Anti-Mouse CD45 #560510 (BD Pharmingen), PerCP-Cy 5.5 Rat Anti-CD11b #550993 (BD Pharmingen), BV605 Rat Anti-Mouse Ly-6C #563011 (BD Horizon), anti-Mouse CD8a PE-Cy7 #60–0081-U025 (TONBO Biosciences), BUV395 Rat Anti-Mouse F4/80 #565614 (BD Horizon), mouse CD1d PBS-57 (an analogue of α-GalCer) (PE-Labeled Tetramer) and mouse CD1d Unloaded (PE-Labeled Tetramer) (NIH Tetramer Core Facility at Emory University). Cells were analyzed on a BD LSRFortessaSORP cytometer analyser and analyzed with free software using https://floreada.io/. Dead cells were excluded from analysis by staining with Live/Dead dye (LIVE/DEAD Fixable Violet Dead Cell Stain Kit #L23105 (ThermoFisher Scientific) (n = 4 or 5).

### Statistical analysis

Results were analyzed using a 3-way ANOVA, using sex, CD1d-genotype and diet as independent factors. Afterwards, Sidak’s multiple comparison test was run between groups differing by only one of the mentioned factors. All statistical analysis was performed using GraphPad Prism 8 (GraphPad Software, San Diego, CA). Results are expressed as mean ± SEM, a p-value < 0.05 was considered significant. In addition, when needed, to test whether mean difference between two groups is statistically significant the Student’s t test was used, and the resulting p value expressed numerically in the figure.

## Results

### HFCD induced steatohepatitis in male and female mice, being more severe in CD1d^−/−^ male mice

**Fig. 1:**
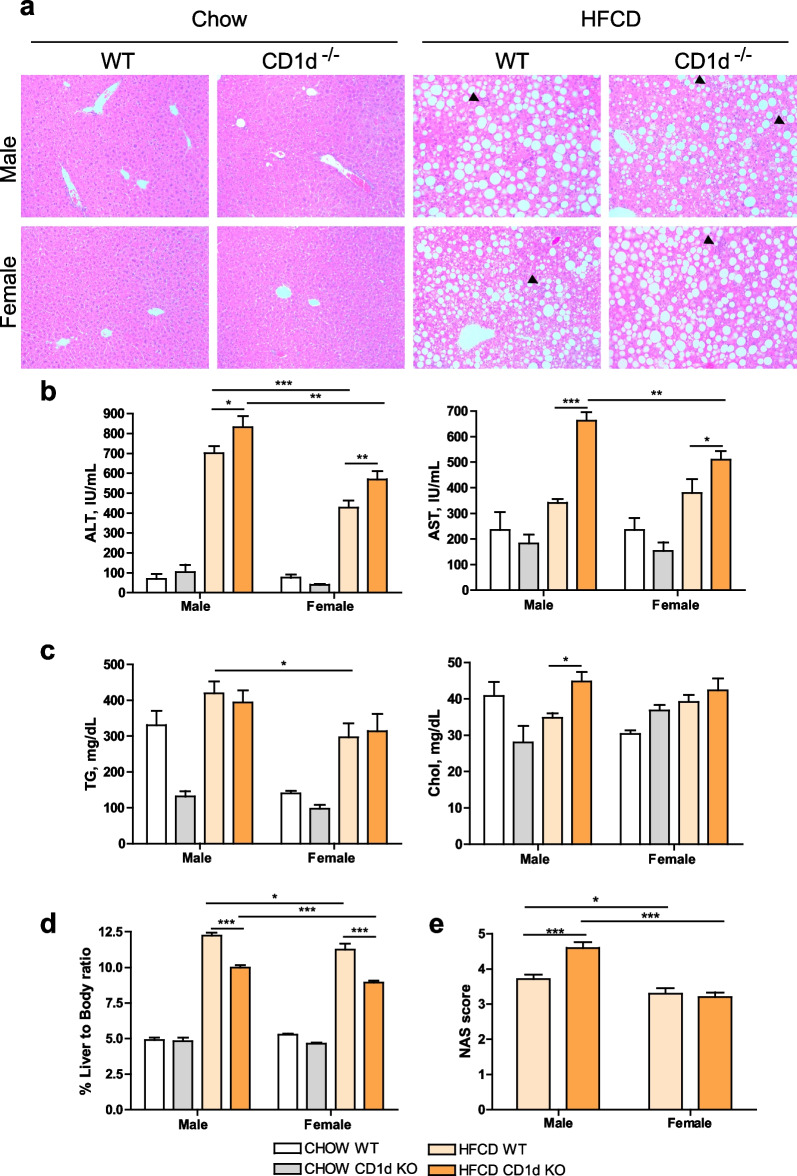
HFCD feeding induces more severe steatohepatitis in males than females, especially in CD1d^−/−^ mice.** a** Representative H&E staining of liver sections, scale bar: 100 µm; **b** serum levels of transaminases, ALT and AST; **c** intrahepatic triglycerides (TG) and cholesterol (Chol) levels; **d** liver-to-body ratio; and **e** NAS score were determined in CHOW, 6 weeks HFCD-fed Balb/c (WT) and CD1d-KO mice, males or females. *n* = 8–10. Data are expressed as mean ± SEM, *p < 0.05, **p < 0.005, ***p < 0.001 vs. corresponding HFCD-groups

The HFCD diet is used as murine model of MASH due to the intrahepatic lipid accumulation triggered by deficiency of choline, essential for liver VLDL (very low-density lipoprotein) particles synthesis and triglycerides export [46]. Thus, we first evaluated hepatic parenchyma morphology by H&E staining and liver damage by transaminase analysis in serum after HFCD diet feeding for 6 weeks. As observed in Fig. 1a, HFCD feeding induced similar macrovesicular steatosis, defined as a single large fat droplet occupying most of the cytoplasm of the hepatocyte, and some aggregates of inflammatory cells (indicated by black arrowheads), in all groups analyzed. For better visualization of the histological findings see Additional file 1. Transaminases, ALT and AST, were increased upon HFCD in all groups, but were significantly elevated in male mice as compared to female mice (Fig. 1b). Moreover, CD1d^−/−^ mice had greater liver damage than their male or female Balb/c counterpart, although it did not reach significance. Regarding lipid composition, we observed the effect caused by the consumption of the HFCD diet with a statistically significant increase in the accumulation of triglycerides in the liver of the HFCD groups vs CHOW groups. Interestingly, males stored more triglycerides than females in the liver, and cholesterol was significantly enhanced in CD1d^−/−^ male mice, as compared to Balb/c male mice after HFCD (Fig. 1c). Of note, liver-to-body ratio was greater in males fed HFCD as compared to females with the same genotype; and among genotypes Balb/c mice had increased liver-to-body ratio than CD1d^−/−^ mice (Fig. 1d). These differences in the ratio liver/body weight were mainly due to the increased liver mass observed in male mice, as compared to females, and to increased liver mass of Balb/c fed HFCD compared to CD1d^−/−^ mice fed HFCD, regardless of the sex considered. For individual graphs of body weight and liver weight see Additional file 1, showing also that the absence of NKT cells did not affect body weight in males or females. Interestingly, among phenotypes, a reduced liver/body ratio was observed in general in CD1d^−/−^ mice that cannot be attributed to fat accumulation, but is probably related to the enhanced liver damaged observed in this phenotype. Moreover, the NAFLD activity score (NAS), a system of scoring the features of MASLD developed as a tool to measure changes in MASLD during therapeutic trials [47] (for more information see the methods section), was significantly increased in male CD1d^−/−^ mice fed HFCD (Fig. 1e), as compared to the other groups. Not shown here, in all Chow-fed mice, male or female, the average NAS score was negligible.

### Liver fibrosis is aggravated in male CD1d^−/−^ mice fed HFCD

**Fig. 2:**
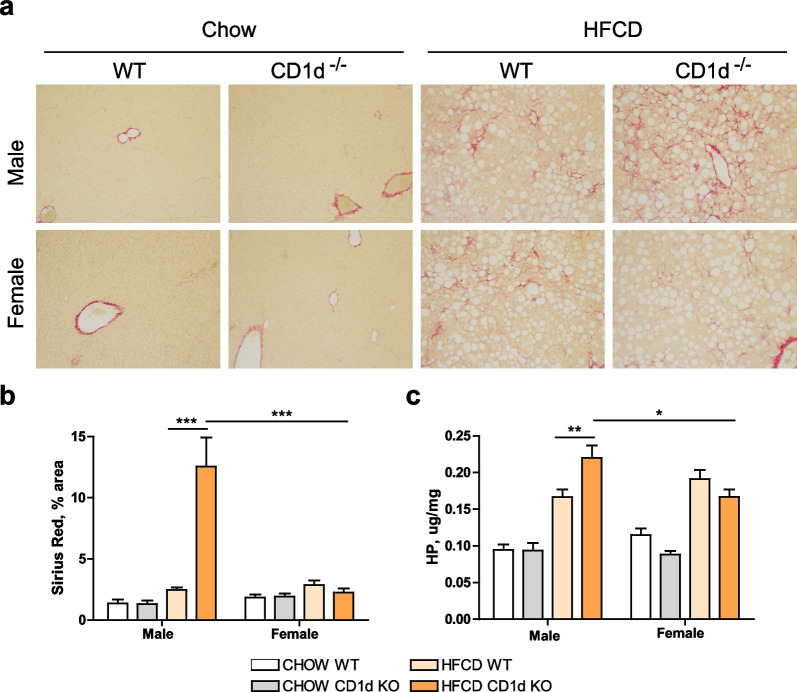
Liver fibrosis is exacerbated in male CD1d^−/−^ mice fed HFCD.** a** representative Sirius red staining of liver sections, scale bar: 100 µm; **b** Sirius red quantification by ImageJ, *n* = 8–10; and **c** hydroxyproline concentration were determined in hepatic tissue in CHOW or 6 weeks HFCD-fed Balb/c (WT) or CD1d-KO mice, males or females, *n* = 8–10. Data are expressed as mean ± SEM, *p < 0.05, **p < 0.005, ***p < 0.001 vs. corresponding HFCD-groups

We next evaluated the presence of fibrosis by first visualizing the red color in collagen fibers using Sirius red staining in hepatic tissue. As shown in Fig. 2a, all HFCD-fed mice, male and female, displayed collagen deposition, with perivenular‐pericellular fibrosis and chicken‐wire patterns characteristic of clinical MASH, without presence of bridging fibrosis. Noticeably, male CD1d^−/−^ mice had the highest presence of collagen among groups.

Imaging quantification of the positive areas for collagen in all HFCD mice, Fig. 2b, indicated similar degree of fibrosis in both sexes, in one hand, and corroborated that male CD1d-/- mice had significantly higher fibrosis than any other group. These results were additionally validated by determining hydroxyproline concentration in hepatic tissue, as shown in Fig. 2c.

**Fig. 3:**
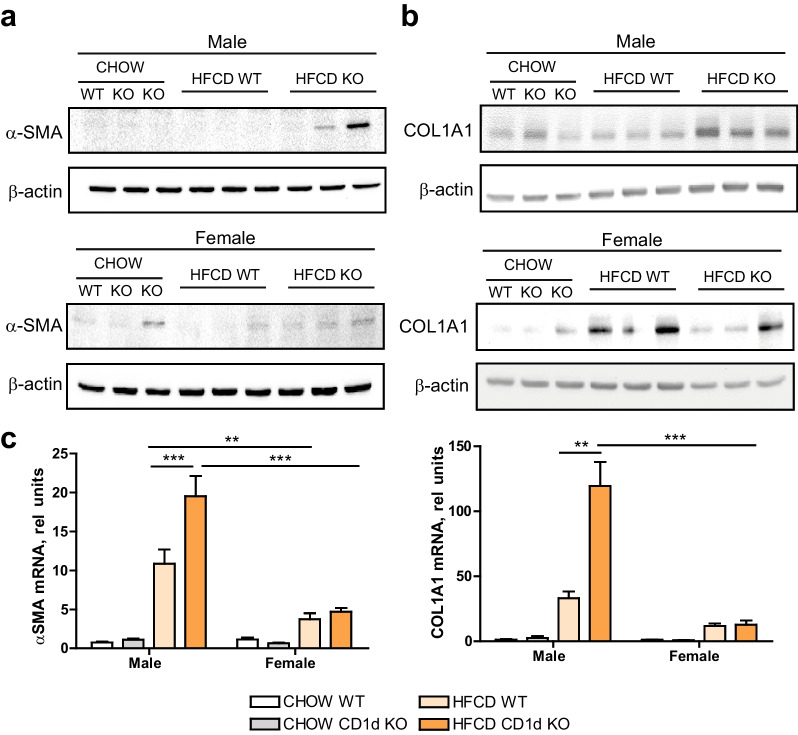
α-SMA and COL1A1 expression in hepatic tissue.** a** Hepatic αSMA, and **b** COL1A1 protein expression were determined in hepatic tissue of 6 weeks fed CHOW or HFCD in Balb/c (WT) or CD1d-KO mice, males or females, by immunoblot, *n* = 1–2 for chow groups, *n* = 3 for HFCD groups. **c** mRNA expression of αSMA and COL1A1, *n* = 5. Data are expressed as mean ± SEM, *p < 0.05, **p < 0.005, ***p < 0.001 vs. corresponding HFCD-groups

Moreover, analysis of HSCs activation markers (α-SMA and COL1A1), main cells responsible for the liver fibrogenic process, as shown by Western blot analysis (Fig. 3a, b) and qPCR (Fig. 3c), indicated that CD1d^−/−^ male mice fed HFCD had increased number of activated HSCs, consistent with the higher degree of fibrosis detected in these livers in Fig. 2.

Of note, mRNA expression of α-SMA and COL1A, Fig. 3c, revealed that male livers, Balb/c or CD1d^−/−^, displayed enhanced levels of these fibrosis markers than female mice, indicative of more active fibrosis in males than females.

### Inflammatory gene expression is enhanced in male mice fed HFCD

**Fig. 4:**
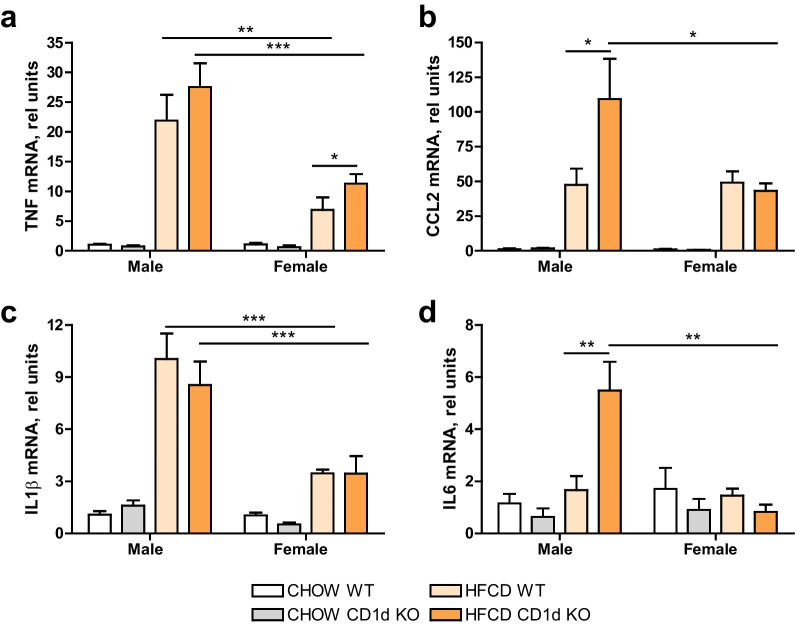
Inflammatory gene expression in hepatic tissue. **a** TNF; **b** CCL2; **c** IL1β; or **d** IL6 mRNA expression were determined by qPCR in hepatic tissue of Chow or 6 weeks HFCD-fed Balb/c (WT) or CD1d-KO mice, males or females. Data are expressed as mean ± SEM, *n* = 5, *p < 0.05, **p < 0.005, ***p < 0.001 vs. corresponding HFCD-groups

When analyzing inflammatory gene expression triggered by HFCD diet consumption, notable differences between both sexes should be highlighted (Fig. 4). In particular, we were able to find inflammatory markers such as TNF, CCL2, IL-1β, or IL6, all of them mainly secreted by Kupffer cells or monocyte-derived macrophages, which were consistently increased after HFCD in male mice compared to female mice, regardless of their genotype.

While in females HFCD induced also an inflammatory response, although to a lesser extent than in male mice, especial mention should be given to IL6 (Fig. 4d) which displayed a higher increase in male HFCD-fed CD1d^−/−^ mice, correlating with disease severity, but was not increased in HFCD-female mice compared to the female chow group.

### NKT-cell deficiency aggravated pro-inflammatory effects of HFCD showing sex distinctive Th1/Th2 polarization

**Fig. 5:**
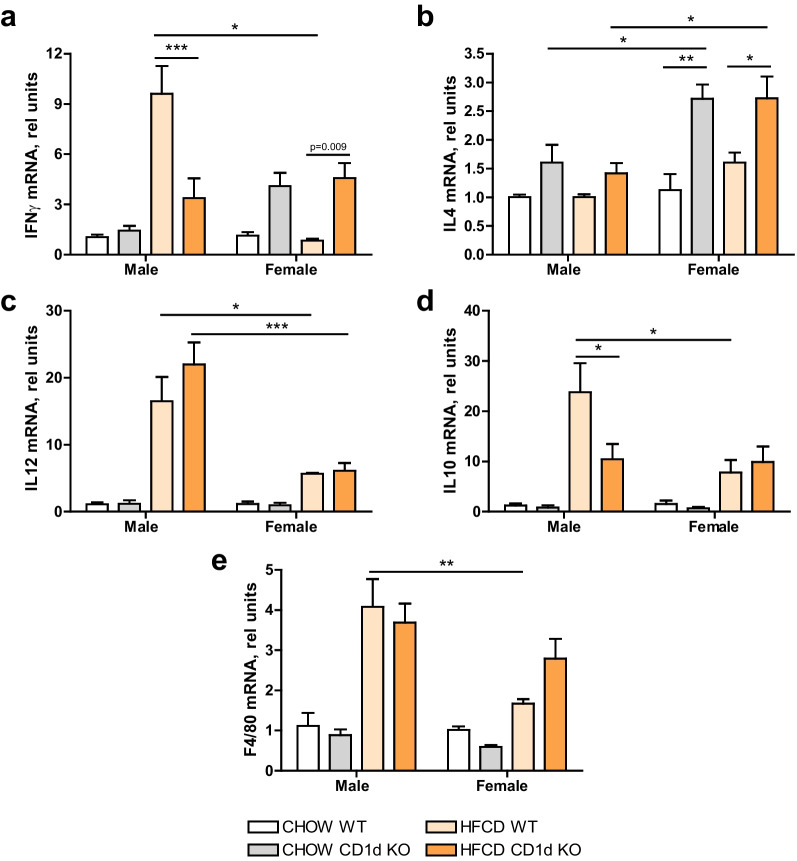
Innate immune gene expression in hepatic tissue. **a** IFNγ; **b** IL4; **c** IL12; **d** IL10; or **e** F4/80 mRNA expression were determined by qPCR in hepatic tissue of Chow or 6 weeks HFCD-fed Balb/c (WT) or CD1d-KO mice, males or females. Data are expressed and mean ± SEM, *n* = 5, *p < 0.05, **p < 0.005, ***p < 0.001 vs. corresponding HFCD-group

We next analyzed whether the absence of NKT cells impacted on typical Th1 (IFNγ and IL12) and Th2 (IL4, IL10) cytokine expression, as shown in Fig. 5. To this aim, we first measured the expression of IFNγ (Fig. 5a). IFNγ was increased in male mice upon HFCD, but its expression was significantly reduced in male CD1d^−/−^ mice as compared to Balb/c mice, consistent with the known role of NKT cells in producing IFNγ [16]. Conversely, when analyzing female mice, we observed on one hand that IFNγ was not increased in Balb/c females after HFCD feeding, and on the other hand, CD1d^−/−^ female mice displayed enhanced levels of IFNγ per se, as compared to chow Balb/c female mice, but IFNγ levels that were not affected by HFCD feeding. These results suggest that IFNγ is differently regulated in males and females. A dissimilar pattern was observed in IL4 (Fig. 5b), that although not enhanced upon HFCD, was found significantly increased in CD1d^−/−^ female mice, but not in male. Thus, implying that NKT-related cytokines, such as IL4 and IFNγ, have a gender-specific behavior independent of MASH.

Moreover, IL12 and IL10 mRNA expression (Fig. 5c, d) were enhanced after steatohepatitis induction, particularly in males. Of note, IL10, cytokine with anti-inflammatory and immunosuppressive properties, was significantly decreased in HFCD CD1d^−/−^ male mice as compared to HFCD Balb/c mice. Finally, macrophage marker F4/80 (Fig. 5e), strongly increased by HFCD in male mice compared to females, but was not affected by NKT deficiency.

### Lack of NKT cells in male mice results in exacerbated neutrophilic and inflammatory macrophages infiltration during steatohepatitis

Given the significant changes in inflammatory and anti-inflammatory gene expression detected after HFCD feeding not only between males and females, but also due to NKT cells deficiency, and to better illustrate how NKTs shape the inflammatory milieu, we performed flow cytometry in the livers to analyze the T cell population and other important immune populations that could result affected by NKTs, mainly macrophages and neutrophils.

**Fig. 6:**
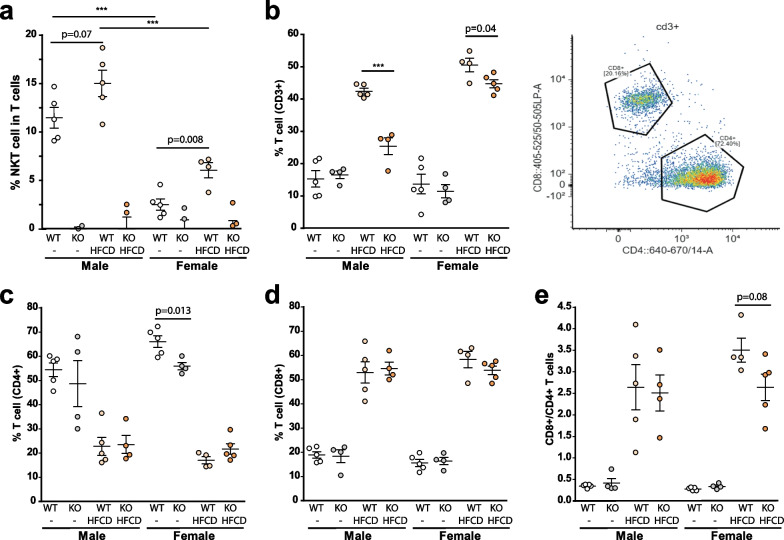
T cell subsets in liver after HFCD in males and females of Balb/c or CD1d-/- mice. The hepatic levels of **a** type I NKT cells (CD3e^+^, Tet-CD1d-PBS-57^+^); **b** % T cells (CD3 +); **c** CD4^+^ T cells; **d** CD8^+^ T cells; or **e** ratio CD8^+^/CD4^+^ T cells were determined by flow cytometry in livers of Chow or 6 weeks HFCD-fed Balb/c (WT) or CD1d-KO mice, males or females. Data are expressed as mean ± SEM, *n* = 4–5, *p < 0.05, **p < 0.005, ***p < 0.001 vs. corresponding compared group. To compare the means between two particular groups the Student’s *t* test will be used, and the resulting p value expressed numerically in the figure

First, we analyzed if the levels of type I NKT cells (CD45^+^ CD3e^+^Tet-CD1d-PBS-57^+^) changed during MASH in our model. While there was an increase in the NKT cells number after HFCD feeding both in male and female Balb/c mice (Fig. 6a). To our surprise, the levels of hepatic NKT cells in female Balb/c mice were significantly and dramatically lower (around 3- to 4-fold) than those observed in male Balb/c mice.

We next analyzed total T cells (CD45^+^CD3^+^) in liver observing a significant increase in this population in MASH both in male and female mice (Fig. 6b). However, there was almost no increase in total T cells in CD1d^−/−^ male mice with steatohepatitis. While we cannot rule out that part of this lack of increase could be due to the absence of NKT-cell expansion characteristic of the mouse model, the magnitude of the numbers indicates that CD1d deficiency compromises also T cell proliferation during steatohepatitis in male mice. Again, the reduction in T cell numbers in CD1d^−/−^ male mice during diet-induced steatohepatitis was more modest in their female counterparts.

When analyzing the T cell subsets: CD4^+^ T cells and CD8^+^ T cells, or the ratio CD8^+^/CD4^+^, in MASH livers, as expected, there was a decrease in CD4^+^ T cells in favor of CD8^+^ cells, as clearly observed in Fig. 6c–e indicative of the chronic inflammatory state in male and female MASH livers. Nevertheless, despite the difference in T cells observed in Fig. 6b, and differences in CD4^+^ and CD8^+^ T cells induced by diet in T cells subsets, we did not observe major differences in T cells subsets due to genotype or sex (Fig. 6c–e). The only exception was the reduced presence of CD4^+^ T cells in the basal state in female CD1d^−/−^ livers as compared to Balb/c females (Fig. 6c).

**Fig. 7:**
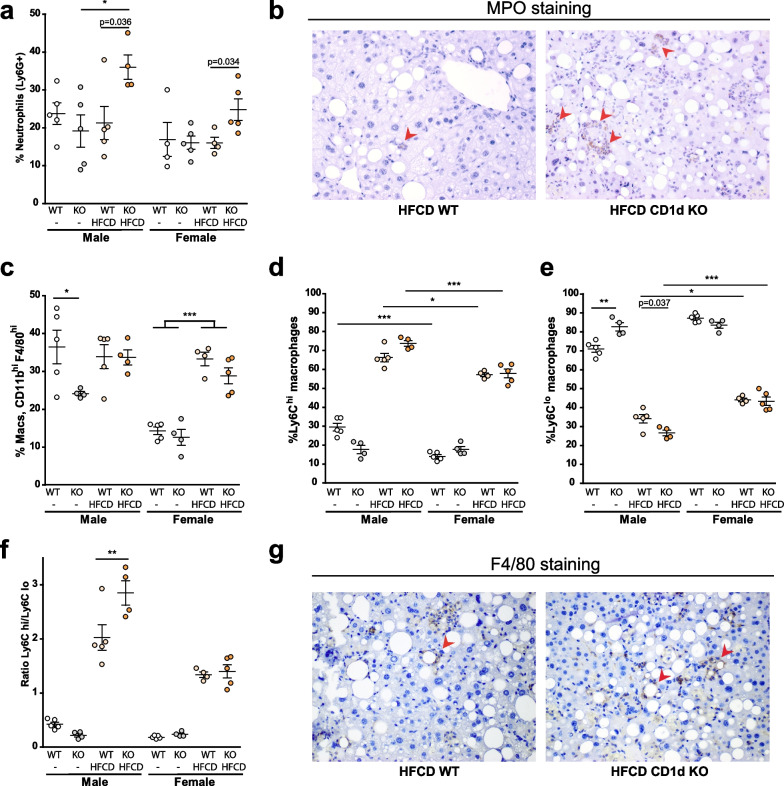
Neutrophils and macrophage subsets in liver in males and females of Balb/c or CD1d^−/−^ mice after Chow or HFCD feeding.** a** Neutrophils (Ly6G^+^); **b** MPO immunohistochemistry (20X), red arrows point to MPO-positive cells in male tissue from sections from 6 weeks HFCD-fed Balb/c (WT) or CD1d-KO mice; **c** macrophages (F4/80^+^, CD11b^+^); **d** Ly6C^hi^ macrophages; **e** Ly6C^lo^ macrophage; or **f** ratio Ly6C^hi^/Ly6C^lo^ macrophages were determined by flow cytometry in hepatic tissue of Chow or 6 weeks HFCD-fed Balb/c (WT) or CD1d-KO mice, males or females; **g** representative F4/80 immunohistochemistry (20x), in 6 weeks HFCD-fed Balb/c (WT) or HFCD-fed CD1d-KO male mice, red arrows indicated hepatic crown-like structures in male tissue sections from HFCD Balb/c (WT) or CD1d-KO mice. Data are expressed as mean ± SEM, *n* = 4–5, *p < 0.05, **p < 0.005, ***p < 0.001 vs. corresponding compared group. To compare the means between two particular groups the Student’s *t* test will be used, and the resulting p value expressed numerically in the figure

We next evaluated how NKT deficiency impacted on immune cells other than T cells. First, we analyzed neutrophils (CD45^+^Ly6G^+^), as observed in Fig. 7a, while neither Balb/c males or females with HFCD show a significant increase in the neutrophil population, CD1d^−/−^ mice fed HFCD displayed significant increased neutrophilic infiltration as compared to the Balb/c groups, this increase in neutrophils was more prominent in male livers. This observation was further validated by analyzing by IHC the presence of myeloperoxidase (MPO)-positive cells or aggregates in MASH tissue in male mice (Fig. 7b). In Balb/c male mice with steatohepatitis there was discrete presence of MPO-positive cells. However, in CD1d^−/−^ male mice with steatohepatitis there was significant presence of MPO-positive cells, as indicated by red arrow-points.

Macrophage levels and phenotype were also analyzed (Fig. 7c–f). Female mice displayed reduced levels of macrophages than male mice in the basal (CHOW) state, Fig. 7c, regardless of the genotype, that significantly increased upon MASH induction. In contrast, in males, CD1d^−/−^ mice had lower levels of macrophages than Balb/c males in the basal state, indicating that CD1d deficiency altered macrophage presence in the liver. In both males and females, MASH induction shifted the population from Ly6C^lo^ predominant in the basal state, to MASH-predominant Ly6C^hi^. Interestingly, the ratio Ly6C^hi^ to Ly6C^lo^ was higher in males than in females with MASH, Fig. 7f, denoting again the lower inflammatory state present in females.

Finally, in CD1d^−/−^ male mice with MASH the ratio Ly6C^hi^ to Ly6C^lo^ was significantly higher than in HFCD-fed Balb/c male mice, consistent with the enhanced inflammatory state observed in these mice by qPCR. Moreover, given the enhanced presence of inflammatory macrophages in males, we also evaluated the presence of hepatic crown-like structures, a histological feature of MASH in mice and humans characterized by the presence of macrophage aggregates surrounding hepatocytes with large lipid droplets [49] observing its presence in Balb/c and CD1d^−/−^ male mice after HFCD (Fig. 7g).

## Discussion

To sum up, our results reveal that the consumption of HFCD diet induces significant liver damage, inflammation and fibrosis in Balb/c mice, with a more pronounced impact in CD1d^−/−^ male mice lacking NKT cells. Moreover, we detected enhanced levels of hepatic iNKT cells in Balb/c mice during HFCD feeding, in both sexes, despite observing that basal hepatic iNKT cell levels in female Balb/c mice were significantly lower than those in Balb/c male. Our model replicates the sex-related differences in disease severity observed in human MASH, as Balb/c female mice displayed moderated MASH features than males. In female Balb/c mice fed HFCD there was decreased presence of inflammatory macrophages (Ly6C^hi^) while displaying a higher presence of reparative macrophages (Ly6C^lo^) as compared to males. Moreover, we observed that IFN-γ and IL-4, two cytokines typically released by NKT cells in response to diverse stimuli, demonstrate distinct gene expression patterns in male and female mice. In all, our data indicate that NKT cells contribute to protect against the development of liver fibrosis and an exacerbated inflammatory response during MASH in Balb/c mice.

The activation of hepatic NKTs seems to contribute both to the onset [34–38, 50, 51] and to the inhibition [39] of inflammation and fibrosis, depending on the etiology of the liver disease being evaluated. In the context of steatohepatitis, several studies with experimental murine models indicate both the accumulation of NKTs in the liver correlating with disease progression [38] and a pathogenic role of these NKTs in its development [33, 37, 52]. However, the majority of these studies share a commonality in using the C57BL/6 mouse strain. In this regard, the severity of steatohepatitis has been demonstrated to change depending on the background of the strain employed [40]. Another pivotal aspect is the lack or sex-perspective in many of these studies, even though the disease displays differential prevalence and severity based on gender in patients [53–55].

Given these considerations, we analyzed the sexual dimorphism in an experimental MASH model induced by a HFCD diet in Balb/c strain animals, along with exploring the role of NKTs within this context. The choice of CD1d^−/−^ mice for this study was made due to their deficiency not only in type I NKT cells (iNKTs), as in prior investigations [39], but also in type II NKT cells, which are less explored in mouse models but more prevalent than iNKTs in the human liver [12].

Our findings reveal that the consumption of an HFCD diet induces significant liver damage in mice, particularly in CD1d^−/−^ animals, with a more pronounced impact in males than females, aligning with the elevated fibrosis and inflammation observed in HFCD-fed male CD1d^−/−^ mice. Thus, our results suggest that the absence of NKTs exacerbates liver damage induced by the MASH diet, irrespective of gender. In line with our observations, Miyagi et al. [39] reported heightened liver damage in Balb/c male mice lacking iNKTs (Jα18^−/−^) and fed a high-fat diet, compared to control mice, within just 5 weeks of dietary intervention. Notably, their study was conducted solely in male mice devoid of type I NKT cells (iNKTs), whereas our investigation explores into the contributions of both type I and type II NKT cells (via CD1d knockout) in both male and female mice. Considering the more prominent presence of type II NKT cells in human livers, as opposed to type I, our utilization of CD1d-knockout mice in this study potentially holds greater relevance to human pathology.

In our model, we observed enhanced levels of hepatic iNKT cells during HFCD feeding, in males and females. Unfortunately, studies investigating NKT cells and their association with MASLD in humans are very limited. Nevertheless, certain research has reported an increase in CD3 + CD56 + cells (NKTs) in the livers of MASLD/MASH patients, primarily through immunohistochemical analysis [38, 56, 57]. Of interest, some of these studies indicate an increase in hepatic NKT cell presence as MASLD progresses to MASH [38, 56]. However, in-depth studies utilizing CD1d tetramers for characterizing NKT presence in MASLD/MASH, and specifically distinguishing gender-related differences, are currently lacking and we hope that our data could fuel such investigations.

The discovery that hepatic iNKT cell levels were markedly lower in female Balb/c mice compared to their male counterparts was entirely unforeseen. In other mouse strains, like C57Bl6/J, hepatic NKT cell levels have been reported to exhibit relatively similar proportions between sexes [58]. However, there is a lack of comparative studies scrutinizing male and female hepatic NKT cells, particularly within the Balb/c strain. Pioneering studies by the Godfrey and [59] and Kronenberg labs [60] have documented hepatic iNKT cell levels in male Balb/c mice and other strains, revealing hepatic iNKT cell percentages around 11% to 22% (respectively, of total T cells), which aligns with our findings in males.

Interestingly, a separate study conducted in Balb/c mice, using the same methodology [59, 60], yielded unexpected results by revealing lower hepatic iNKT cell levels [61], constituting approximately 4% of total T cells. It is worth noting that this study solely focused on female Balb/c mice. Remarkably, the levels found in hepatic iNKT cells in females Balb/c mice are similar to the levels reported in here. Thus, to the best of our knowledge, our report is the first study comparing simultaneously hepatic iNKT cells in male and female mice of the Balb/c strain.

However, the absence of an assessment of type II NKT cell levels is a limitation of our study. Despite this limitation, the lower level of hepatic iNKT cells in females found in our study is consistent with the fact that male mice are more profoundly affected than females during steatohepatitis by the lack of NKT cells, and to the observation that this has a major impact in other inflammatory cell types.

When assessing liver/body weight ratios, HFCD diet-fed mice displayed increased values in both sexes, indicative of the accumulation of intrahepatic lipids and compatible with the enhanced accumulation of triglycerides observed after HFCD, due in one hand to the high fat diet composition of the diet (60%), and to the lack of choline that hampers VLDL exports from the liver [62]. Moreover, the triglyceride accumulation and liver/body ratio were superior in male mice.

Of interest, IFN-γ and IL-4, two cytokines with apparently opposite functions (pro-inflammatory and pro-fibrotic, respectively) and typically released by NKT cells in response to diverse stimuli, demonstrate distinct gene expression patterns in male and female mice. In females, the CD1d^−/−^ groups displayed an elevation in liver gene expression for both cytokines, regardless of the diet, possibly indicating a compensatory mechanism set off by other liver IFN-γ-producing cells (like NK cells, MAIT, or γδ T cells) due to the absence of NKT cells. Conversely, in males, IL-4 gene expression levels remained unchanged across different groups, while IFN-γ expression notably surged in the HFCD Balb/c group (in comparison to the HFCD CD1d^−/−^ group and the control group). This increase underscores the role of NKT cells in IFN-γ secretion and the protection against diet-induced steatohepatitis in male mice, since the HFCD CD1d^−/−^ group exhibited a greater inflammatory response even in the absence of the IFN-γ generated by NKTs.

In agreement with previous studies in male Balb/c mice [39], we observe that NKT cells contribute to protect the liver against the development of liver fibrosis and an exacerbated inflammatory response during MASH. In contrast, investigations on the C57BL/6 mouse strain reveal a different facet, implicating NKT cells in a pro-fibrotic and pro-inflammatory capacity in diet-induced steatohepatitis [35–38]. Deciphering the cause of this discrepancy, one must consider the polarization of the immune system. While the C57BL/6 mice tend towards a Th1-polarized response, Balb/c mice lean towards a Th2-immune response [63]. Importantly the differential behavior of NKT cells in the Balb/c and C57Bl6/J strains seems not to be specific for MASH. A systematic study performed in the context of malaria infection reported that CD1d-restricted natural killer T (NKT) cells can contribute to either protection against or susceptibility to malaria depending on the host genetic background [64]. Investigating the role of CD1d-restricted NKT cells in terms of mortality percentage and infection-associated histological markers, on the Balb/c background NKT cells seem protective, while on the C57BL6 background were not, since CD1d^−/−^ C57BL6 mice experienced partial protection against disease [64]. These data, consistent with our results, support that the behavior of NKT cells depends on the immune makeup of the strain which has been proved critical in determining the outcome in diseases such as obesity and diabetes [65].

In concordance with human pathology, our diet-induced steatohepatitis rodent model mimics sex-related differences in disease severity. Specifically, male subjects exhibited heightened liver damage, hepatic inflammation, and more pronounced steatosis, consistent with previous research [44, 45]. Elevated expression of fibrogenic genes (α-SMA, COL1A1) in males further accentuated this disparity. Similarly, in humans, it is acknowledged that women excel in resolving inflammation and limiting chronic inflammation more effectively than men [66]. In addition, the established influence of estrogens in shaping the female immune system and safeguarding against MASH should be noted [41–43]. Remarkably, CD1d^−/−^ animals displayed amplified fibrosis in males compared to females, indicating a more active fibrotic process. Our cytometry data support this assessment by identifying a higher proportion of inflammatory macrophages (Ly6C^hi^/Ly6C^lo^) in HFCD CD1d^−/−^ male mice, with potential involvement in driving fibrosis [67].

Furthermore, flow cytometry data underscore marked distinctions between females and males. Specifically, females exhibit fewer inflammatory macrophages (Ly6C^hi^), even in their basal state, while displaying a higher presence of reparative macrophages (Ly6C^lo^). In our model, female mice demonstrate subtle NKT-dependent protection against HFCD consumption, maybe due to their relatively lower levels of iNKT cells. Changes in liver damage (ALT levels) and the immune pattern, displaying increases levels of neutrophils and a growing proportion of inflammatory macrophages, suggest that over an extended period NKT-induced protection might assume a significant role in female mice confronted with heightened damage and inflammation.

Consistent with this view, our preliminary data from a liver cancer model (Cuño-Gómiz et al., unpublished results), involving diethyl nitrosamine (DEN) injection in combination with HFCD diet confirms this notion. In fact, our initial findings reveal a significant surge in alpha-fetoprotein (AFP), a biomarker for hepatocellular carcinoma (HCC), in CD1d-deficient female mice serum compared to Balb/c female mice following 8 months of treatment with DEN + HFCD (9.134 ± 1.957* µg/ml of AFP in CD1d-KO female mice, n = 10, vs. 3.602 ± 0.641 µg/ml of AFP in Balb/c female mice, n = 7, p < 0.01). While these observations are promising, further investigation is essential considering the complex interplay of factors involved. Reliable animal models that accurately emulate human pathology are necessary for advancing scientific understanding.

## Perspectives and significance

Our findings imply that hepatic NKT cells play a role in mitigating inflammation during MASH progression in male Balb/c mice by modulating the immune environment towards a more favorable state for inflammation control. In female Balb/c mice, the inherent protection maybe conferred by estrogens and the female immune response appears effective in managing early MASH with minor influence from NKT cells. In this sense, our results may provide a broader perspective on the sex-dependent influence of NKT cells in other inflammatory diseases, beyond its protective role in MASH or in liver tumorigenesis.

### Supplementary Information

**Additional file 1. ** HFCD feeding induces more severe steatohepatitis in males than females, especially in CD1d^−/−^ mice. **a** Representative H&E staining of liver sections, scale bar: 100 μm; **b** body weight; and **c** liver weight of 6 weeks HFCD-fed Balb/c (WT) and CD1d-KO mice, males or females. n = 8–10. Data are expressed as mean ± SEM, *p < 0.05, **p < 0.005, ***p < 0.001 vs. corresponding HFCD-groups.

## Data Availability

The datasets generated and/or analyzed during the current study are available from the corresponding authors on reasonable request.
