# Supplementary figures and images for: Sex-based differences in natural killer T cell-mediated protection against diet-induced steatohepatitis in Balb/c mice
Source: Biol Sex Differ. 2023 Nov 14;14:85. doi: 10.1186/s13293-023-00569-w (PMC10644614; doi:10.1186/s13293-023-00569-w)

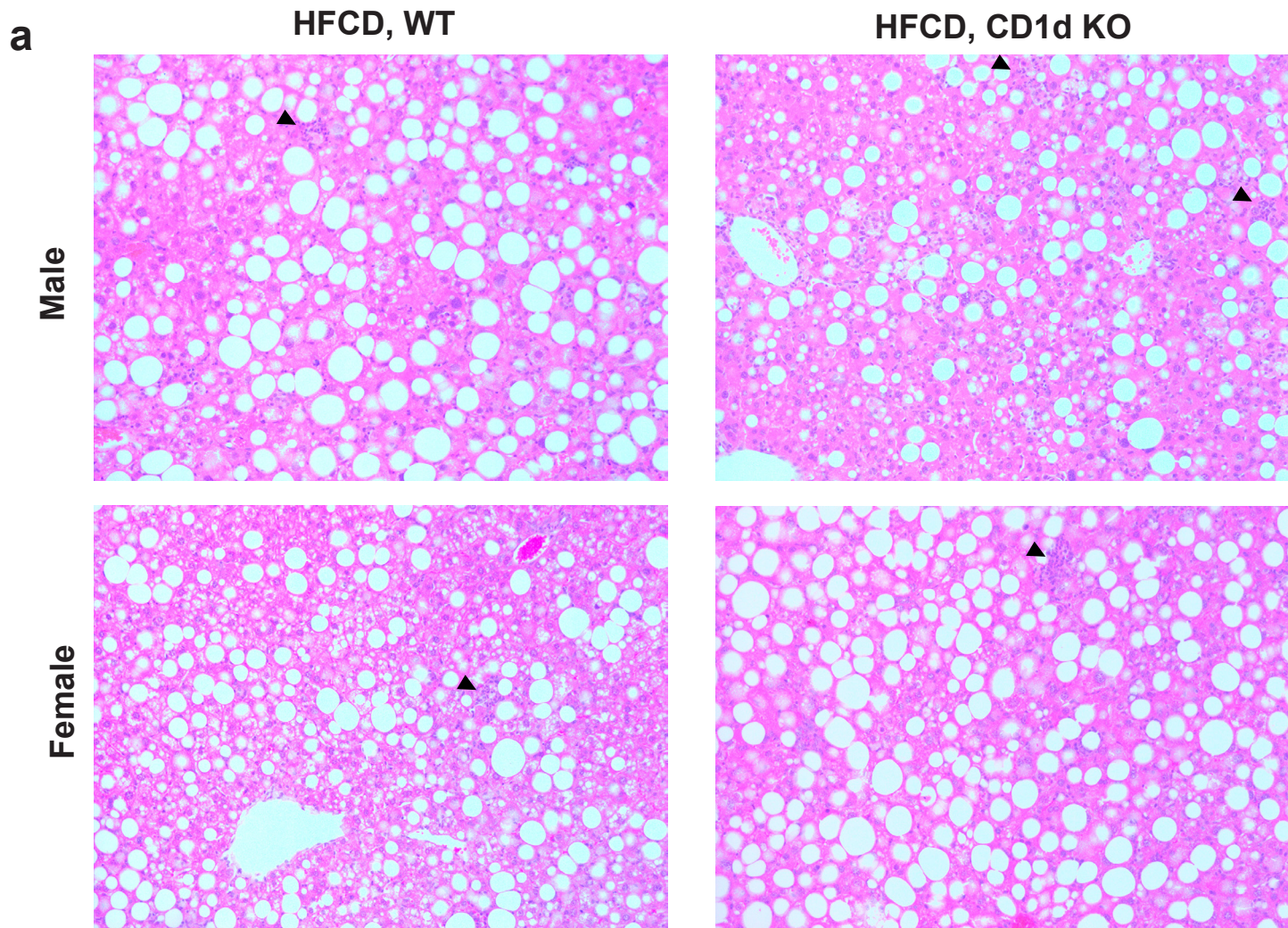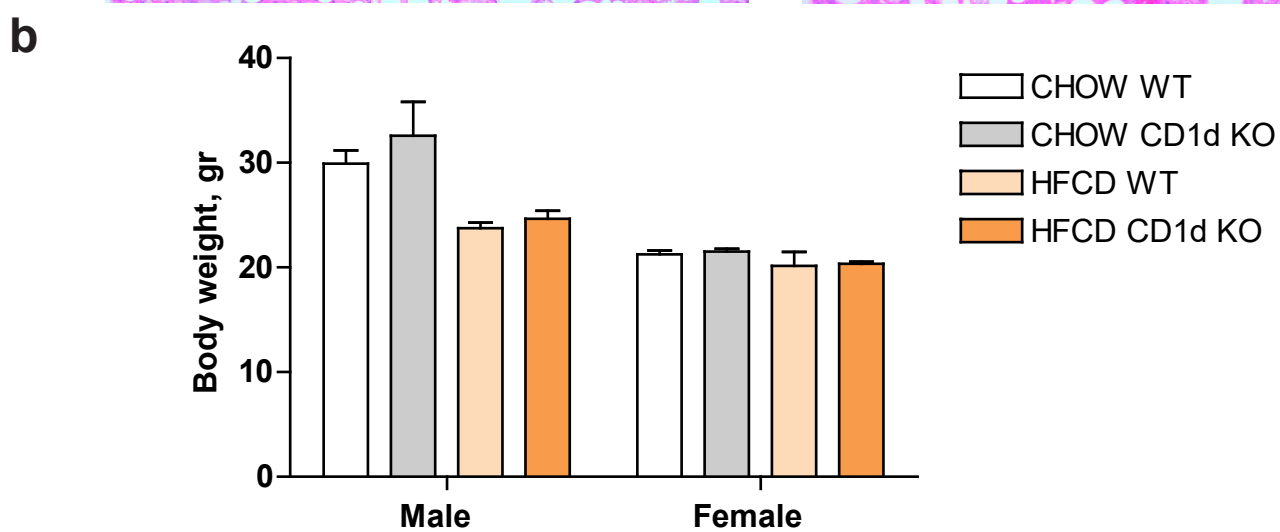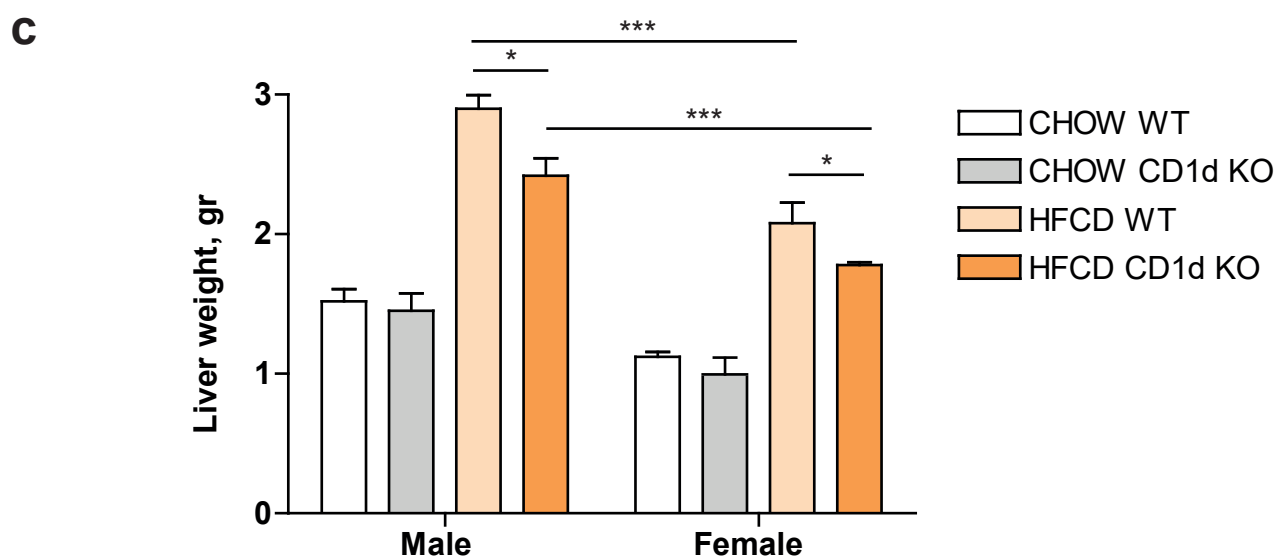

Supplement: Supplementary file 1 — Additional file 1. HFCD feeding induces more severe steatohepatitis in males than females, especially in CD1d−/− mice. a Representative H&E staining of liver sections, scale bar: 100 μm; b body weight; and c liver weight of 6 weeks HFCD-fed Balb/c (WT) and CD1d-KO mice, males or females. n = 8–10. Data are expressed as mean ± SEM, *p < 0.05, **p < 0.005, ***p < 0.001 vs. corresponding HFCD-groups. [file 13293_2023_569_MOESM1_ESM.pdf]
